# Ununited Fractures

**Published:** 1828-10

**Authors:** 


### HOSPITAL REPORTS,

*(Principally condensed from various Periodical Publications.)*

### UNUNITED FRACTURES.

*Cases of Ununited Fractures, treated at the* ROYAL INFIRMARY OF EDINBURGH.*

AMONGST the numerous cases of fracture which always constitute a large share of our materials for clinical observation, you have had an opportunity, during the present course, of seeing two remarkable cases; in one of which the process of reunion has been excessively slow, and in the other altogether suspended.

The first of these patients, Donald Clark, aged seventy-two, was admitted on the 26th March under my care, having sustained a fracture of the leg, described in the journal as follows:

"The tibia is fractured about one inch and a half below its tubercle; and again, two inches lower down, the intermediate portion of bone is loose and moveable; the fibula is broken nearly opposite to the lower fracture of the tibia. Injury was the consequence of his leg having been pressed between a coal-cart and a wall.

"Limb was placed on M'Intyre's splint, with a pasteboard splint on either side, firmly confined with a roller.

"May 10.—Upper fracture has united, but lower one crepitates distinctly when the limb is moved. To have beef, porter, &c.

"June 1.—No crepitus; but still great mobility at the seat of the lower fracture.

"26.—To sit up every day, and walk upon crutches.

"July 12.—Mobility has decreased considerably. Dismissed relieved."

This man has been visited by the house-surgeon today, who reports that the union of the tibia, although still incomplete, has of late been advancing by slow degrees. The fractured extremities of the bone are, and have all along been, in perfect apposition; the intermediate portion between the two fractures has preserved its vitality, and I can see no circumstance which should have prevented the more speedy reunion of the lower fracture, except the defects of a constitution which has all the appearance of having been impaired by hard labour, and the accession of a premature rather than an extreme old age. The limb was firmly secured in pasteboard splints, and the patient dismissed with an injunction to move as much as possible with the aid of crutches, for the purpose of exciting action in the fractured part of the limb.

This is a case, in my opinion, ill calculated for the adop-

* From Dr. BALLINGALL's Clinical Lecture, July 1828.

tion of any of those expedients which surgery holds out for the treatment of cases of this kind. When union has been retarded, as in this instance, from a general defect in the habit, we have no reason to expect that such expedients would succeed: the man's constitution cannot be improved by four months' confinement in the hospital.

The other case of ununited fracture is that of Thomas Christie, aged forty-one, a patient of Mr. Liston's, who was admitted on the 9th of June, and stated "that six weeks ago, when at work down a pit, a piece of wood fell and struck him upon the arm, which was fractured. A good deal of swelling and inflammation of the arm succeeded, for which he was once bled from the arm, and at different times had leeches applied, to the number of eighty. Three weeks after the accident, splints were applied, which he says were repeatedly obliged to be removed, on account of the swelling and inflammation.

There is a transverse fracture of the humerus four inches above the elbow. The ends of the bone overlap each other, the lower portion going in front: not the slightest union has taken place, and the ends of the bone cannot be brought into apposition when extension is used; general health good. The arm, since the accident, has been kept in a bent position."

On the 12th, "the forearm was tightly bandaged with a flannel roller; a pasteboard splint was applied on the outer and inner side of the arm, and a wooden splint applied firmly on the outside over all." The patient was ordered a beef-steak and a bottle of porter daily; and the splints were continued on the arm until the 30th, when the following report is entered:

"No union has taken place; the bones are as loose, seem to have the same motion, and to remain at as great a distance from each other. Mr. Liston removed a portion of the broken extremity of each bone, by cutting them somewhat obliquely. A splint was applied on the inner side of the arm, and which supported the whole of the forearm; a short splint was applied on the outside. Complains of pain at elbow; passed a quiet night; not much sleep; bowels not open; tongue rather loaded; pulse ninety-six; skin cold; not much pain in the wound."

A considerable degree of swelling and tension followed the operation, which rendered it necessary to remove the outer splint. A great part of the wound at either extremity healed by the first intention, the centre of it being kept open for some time by the discharge of a collection of matter which formed over the region of the biceps muscle. The whole wound is now nearly cicatrised, and the case wore a very promising appearance until within these few days, when the patient met with a most untoward accident by falling out of bed during sleep, by which the process of reunion has no doubt been disturbed, but it is to be hoped will not ultimately be prevented.

In speaking of this case, I noticed a number of others in which this operation had been practised with various degrees of success: I fear, however, that I omitted to mention, or to give you a reference to two interesting cases treated some years ago in this house, one by the late Dr. Wardrop, and the other by my colleague Dr. Inglis. Of these two cases you will find the particulars detailed in the first volume of the Edinburgh Medical and Surgical Journal; and what renders them the more remarkable is, that in both instances union was procured, although the fracture existed in the one case in the forearm, and in the other in the leg; situations in which Boyer and other eminent surgeons had altogether discountenanced the performance of this operation.

---

